# DUOX1 Gene Missense Mutation Confers Susceptibility on Type 2 Amiodarone-Induced Thyrotoxicosis

**DOI:** 10.3390/ijms24044016

**Published:** 2023-02-16

**Authors:** Olga Biakina, Yulia Mitina, Daria Gognieva, Marina Axenova, Alexandra Ermolaeva, Afina Bestavashvili, Valentin Fadeev, Abram Syrkin, Philipp Kopylov

**Affiliations:** 1 Department of Cardiology, Functional and Ultrasound Diagnostics of N.V. Sklifosovsky Institute for Clinical Medicine, I.M. Sechenov First Moscow State Medical University (Sechenov University), 119991 Moscow, Russia; 2 Department of Anatomy and Physiology, School of Biomedical Sciences, University of Melbourne, Melbourne 3010, Australia; 3 Murdoch Children’s Research Institute, Melbourne 3052, Australia; 4 World-Class Research Center «Digital Biodesign and Personalized Healthcare», I.M. Sechenov First Moscow State Medical University (Sechenov University), 119991 Moscow, Russia; 5 Federal State Organization “Centre for Strategic Planning and Management of Biomedical Health Risks”, Ministry of Health of the Russian Federation, 119121 Moscow, Russia; 6 Department of Endocrinology No. 1 of N.V. Sklifosovsky Institute for Clinical Medicine, I.M. Sechenov First Moscow State Medical University (Sechenov University), 119991 Moscow, Russia

**Keywords:** amiodarone-induced thyrotoxicosis 2 type, genetic markers, DUOX1 gene

## Abstract

Possible triggers and genetic markers involved in pathogenesis of amiodarone-induced thyrotoxicosis (AIT) or amiodarone-induced hypothyroidism (AIH) are currently unknown. This study aimed to analyze the association between polymorphisms in the genes involved in thyroid hormones biosynthesis and metabolism. Thirty-nine consecutive patients with confirmed type 2 amiodarone-induced thyrotoxicosis were enrolled; 39 patients on the same therapy for at least 6 months without thyroid pathology were included as a control group. A comparative study was carried out to determine the distribution and genotypes of polymorphic markers of the (Na)-iodide symporter (NIS) genes (rs7250346, C/G substitution), thyroid stimulating hormone receptor (TSHR) (rs1991517, C/G substitution), thyroid peroxidase (TPO) (rs 732609, A/C substitution), DUOX 1-1 (C/T substitution), DUOX 1-2 (G/T substitution), DUOX 1-3 (C/T substitution), glutathione peroxidase 3 (GPX3) (C/T substitution), glutathione peroxidase 4 (GPX4) (C/T substitution). Statistical analysis was performed using Prism (Version 9.0.0 (86)). This study showed that the risk of AIT2 is 3.18 times higher in the G/T of the DUOX1 gene carriers. This study is the first report of genetic markers associated with amiodarone-related adverse events conducted in humans. The obtained results indicate the necessity for a personalized approach to amiodarone administration.

## 1. Introduction

Amiodarone is a commonly prescribed class 3 antiarrhythmic drug and one of the most effective medications for managing both atrial and ventricular rhythm disturbances [1]. The amiodarone molecule shares structural similarities with thyroxine. It contains two iodine atoms, amounting to 37 mg of iodine per 100 mg of amiodarone, of which 10% is de-iodinated to yield free iodine [2]. 

Unfortunately, the physiological synthesis of thyroid hormones, thyroxine (T4) and 3,5,3’-triiodothyronine (T3) may be adversely impacted in a euthyroid patient following amiodarone administration. Thyroid abnormalities have been noted in up to 14–18% of patients receiving long-term amiodarone therapy. However, a meta-analysis suggested that with lower doses of amiodarone (150–330 mg), thyroid dysfunction is 3.7%; test findings show effects ranging from abnormal thyroid function to overt thyroid dysfunction, which may be either amiodarone-induced thyrotoxicosis (AIT) or amiodarone-induced hypothyroidism (AIH) [3,4,5,6].

The prevalence of AIT in the United States is 3% and the prevalence of AIH is 22%. The relative prevalence of the two forms of AIT is unknown. In Russia, AIT’s development frequency is estimated to be around 5.8–15.8%; AIT2 prevalence is not known [7,8]. 

The possible triggers, genetic markers and associations involved in its pathogenesis are unknown. However, involvement of mechanisms different from the iodine overload were considered: direct toxicity showed in amiodarone-induced pulmonary damage, reactive oxygen species (ROS) generation, altered calcium ions regulation, drug-induced lysosomal lipidosis and disruption of mitochondrial function [9,10].

In this study, we aimed to analyze the association of polymorphisms in the genes involved in thyroid hormones biosynthesis and metabolism with AIT and AIH.

## 2. Results

### 2.1. Patient Characteristics

Patient characteristics are listed in Table 1. 

A homogeneous population of patients was recruited into different study arms. Groups significantly differ by the hormone levels, while the other baseline characteristics were either the same (e.g., the total number of patients, sex distribution) or did not display statistical significance (e.g., median age, the serum concentration of amiodarone).

Both our groups consisted of 39 patients. Twenty patients in each group were males. The median age was 67.6 ± 7.9 years in the study arm and 69.7 ± 8.1 years in the control arm (*p*-value—0.0934; Fisher’s exact test).

### 2.2. Associations between Genotypes and Clinical Outcomes

Eight single nucleotide polymorphisms (SNPs) of genes involved in thyroid hormones biosynthesis and metabolism were genotyped in patients from both study arms. The SNP genotype, gene information, genotype frequency and the associated clinical response are shown in Table 2. Binary logistic regression was also performed for genotype distribution: genotypes containing the minor allele were compared to the ancestral homozygous genotype. Results are shown in Table 3. As three different SNPs related to DUOX1 gene were investigated, they were labeled with consecutive numeric labels for convenience. The G/T genotype of DUOX1-2 (rs17595239) was significantly associated with type 2 amiodarone-induced thyrotoxicosis (AIT2) development (*p* = 0.0063, q = 0.0493, Cochran—Armitage trend test with Bonferroni— Šidák correction). The same association was revealed using binary logistic regression (*p* = 0.024) with an odds ratio of 3.18. 

No significant association between genotypes and clinical outcomes was observed for the remaining SNPs.

## 3. Discussion

The influence of SNP of genes involved in thyroid biosynthesis and metabolism on the outcomes of type 2 amiodarone-induced thyrotoxicosis was assessed. Interestingly, our study showed that patients with the G/T genotype of DUOX1 gene (rs17595239) are 3.18 times more likely to develop AIT2, which did not demonstrate an association with any disease in genome-wide association studies previously. 

DUOX1, also known as ThOX1, a member of the NOX/DUOX family, is part of a protein complex located on the apical membrane of thyroid follicular cells [9]. This complex consists of an iodide transporter (encoded by NIS gene), thyroperoxidase (TPO gene) and another member of the peroxide generating system, DUOX2. Under the physiological conditions, hydrogen peroxide availability is a rate-limiting condition for the thyroid hormones biosynthesis; H2O2 plays the final electron acceptor’s role [10]. Ultimately, the crucial role of DUOX1 in human thyroid function was demonstrated in patients with congenital hypothyroidism (CH): two heterozygous missense mutations in DUOX1 and DUOXA1 in two patients can cause CH through disrupting the coordination of DUOX1 and DUOXA1 in the generation of H2O2. The two missense mutations of DUOX1 exhibited partial loss (R1307Q, R56W) of H2O2-generating activity; all of them were documented in patients harboring these mutations anticipating a causal link with the impaired iodide organification [11]. It was also previously shown that the G/G genotype of DUOX1 (rs2467825, alleles-A/G) was protective of diffuse euthyroid goiter in the Moscow population [12].

The expression of DUOX1 was also detected on the lung and intestine surface epithelia. It is shown to increase under interleukin 4 stimulation, likely playing an essential role in the process of innate immunity as well [13,14]. Nevertheless, DUOX1-mediated ROS production (as ROS production in general) has to be controlled to prevent a nonspecific activation responsible for excessive damage to the host tissue, e.g., during chronic inflammation [13].

The AIT2 is a form of hyperthyroidism characterized by actual destruction of thyroid tissue, inflammation, fibrosis and ultimately depletion of the gland’s vascularity [15]. Our study indicates the possible mechanism of the tissue disruption: amiodarone-DUOX1 mediated uncurbed ROS generation.

However, our study had several limitations. First, the sample size was small due to low availability of these patients. Second, there was only a limited number of SNPs tested. Third, the clinically attainable level of amiodarone was limited, as we consciously omitted the possible involvement of cytochrome P 450 isoforms into amiodarone metabolism. We have not determined N-desethyl-amiodarone to amiodarone ratio and the influence of epidemiological parameters and patients’ characteristics on the clinical outcome development. Nevertheless, we demonstrated a clinically relevant and significant association between DUOX1 SNP and AIT2 development.

Furthermore, our ongoing research is focused on determining the combined influence of cumulative amiodarone dose, SNP and other epidemiological factors in a larger cohort of patients. The results will be demonstrated in subsequent publications.

## 4. Materials and Methods

### 4.1. Statistical Analysis

Statistical analysis was performed using Prism (Version 9.0.0 (86)). Clinical responses among the genotypes were analyzed using the Cochran—Armitage test or chi-square test with Bonferroni—Šidák correction for multiple hypothesis testing. Patients’ baseline characteristics were analyzed using Student’s t-test for continuous variables with normal distribution and the Mann—Whitney U test for non-normally distributed variables. Age- and gender-adjusted binary logistic regression was used to identify predictors of the disease development. A *p*-value of less than 0.05 was considered significant for all analyses.

### 4.2. Study Population

Between September 2013 and December 2016, a total of 51 consecutive patients with clinically and functionally confirmed type 2 amiodarone-induced thyrotoxicosis were treated on the clinical bases of the Sechenov University (University Clinical Hospitals № 1 and 2). Among these 51 patients, we enrolled 39 registered in the hospitals mentioned above. The inclusion criteria were the following: (1) history of amiodarone treatment; (2) a serum concentration of amiodarone at 0.5–2.5 mg/mL; (3) decreased levels of thyroid-stimulating hormone (TSH), increased concentration of free thyroxine (T4), free triiodothyronine (T3); (4) an decrease of 99mTc-pertechnetate accumulation and thyroid uptake of less than 1% according to scintigraphy. The remaining 12 patients were excluded from the study according to the exclusion criteria: (1) any proven thyroid disease prior to amiodarone treatment; (2) administration of lithium drugs, glucocorticoids, phenytoin, interferon, iron supplements, estrogens, somatostatin analogs; (3) pregnancy and lactation; (4) severe comorbidities; (5) mental disorders affecting patient compliance. Thirty-nine patients receiving amiodarone treatment for at least six months exhibiting no thyroid pathology were included as a control arm.

Participants have provided written informed consent and all experimental methods were in accordance with the 1975 Declaration of Helsinki. This study was approved by the Review Board of the Sechenov University. All procedures were performed in accordance with the ethical principles for medical research.

### 4.3. Thyroid Gland Functional Tests

The determination of the levels of TSH, free T4 andT3 in the serum was performed by the method of enhanced chemiluminescence with the Architect automatic analyzer (Abbott Laboratories, Chicago, Illinois, USA). The basal levels of TSH equal to 0.4–4.0 μIU/mL, free T3—3.5–6.5 pmol/L, free T4—11.5–23.2 pmol/L were considered as normal.

Thyroid gland scintigraphy with 99mTc-pertechnetate was carried out in the radionuclide diagnostic department of the Sechenov University using a GE 400T rotating gamma camera (General Electric, Boston, MA, USA).

### 4.4. SNP Selection

SNPs with putative functional effects were selected. A comparative study was carried out to study the distribution and genotypes of polymorphic markers of the NIS genes (rs7250346, C/G substitution), TSHR (rs1991517, C/G substitution), TPO (rs 732609, A/C substitution) DUOX 1-1 (C/T substitution), DUOX 1-2 (G/T substitution), DUOX 1-3 (C/T substitution), GPX3 (C/T substitution), GPX4 (C/T substitution). We have deliberately chosen the SNPs on different chromosomes or not in a linkage disequilibrium (r2, D’ = 0). R2 and D’ were calculated using LDlink (https://ldlink.nci.nih.gov/). 

### 4.5. Deoxyribonucleic Acid (DNA) Genotyping 

Isolation of genomic DNA from whole blood was carried out using the K-Sorb reagent kit for DNA isolation on microcolumns by the manufacturer’s instructions (Syntol joint-stock company (JSC)).

Genotyping of the studied polymorphisms was carried out by real-time polymerase chain reaction (PCR) using primers and TaqMan-probes (Syntol JSC). A list of sequences of primers, probes and annealing temperature Ta for the related polymorphisms is presented in Supplement 1.

The total volume of the reaction mixture was 25 μL. The mixture contained 20–50 ng of isolated DNA, a pair of primers at a concentration of 300 nM each and a corresponding pair of TaqMan probes at a concentration of 100 nM each, 200 μM deoxynucleoside triphosphate, ammonium sulfate amplification buffer with 2 mM MgCl_2_, Hot Start Taq DNA polymerase—0.5 units of the act. (Limited liability company “SibEnzyme”).

PCR was performed using a StepOnePlus real-time amplifier (Applied Biosystems, USA). We used the following parameters: initial denaturation for 3 min at 94 °C; then 40 cycles, including denaturation at 94 °C for 10 s., annealing of primers and subsequent elongation at the above Ta for 30 s. with the registration of the carboxyfluorescein (FAM) and rhodamine 6G (R6G) fluorescent signal at each amplification step.

Verification of genotyping of the studied polymorphisms was performed by restriction fragment length polymorphism. PCR of the corresponding loci was performed using the primers and annealing temperatures (Ta) presented in Supplement 2 and the supplementary Table S1.

## 5. Conclusions

At present there are no data regarding the pharmacogenomics of amiodarone. This study is the first report of genetic markers associated with amiodarone-related adverse events conducted in humans. It investigated the contribution of gene polymorphism to the development of type 2 amiodarone-induced thyrotoxicosis and showed that the risk of AIT2 is 3.18 times higher in the G/T of the DUOX2 gene carriers. The obtained results indicate the necessity for a personalized approach to amiodarone administration for the most effective and safe usage of this medication.

## Figures and Tables

**Table 1:** The general characteristics of patients enrolled in this study. *p-values are estimated by Fisher’s exact test.

Characteristics	Study Group	Control Group	*p*-Value
Total Patients	39	39	
Median Age, years	67.6 ± 7.9	69.7 ± 8.1	0.0934
Males (%)	20 (51%)	20 (51%)	
Never Smoked (%)	35 (90%)	36 (92%)	
Serum AmiodaroneConcentration, mg/mL	1.81 ± 0.54	1.73 ± 0.59	0.752
Hormones Levels	TSH 0.12 ± 0.06 μIU/mL	TSH 3.05 ± 1.8 μIU/mL	0.023
	T3 9.4 ± 1.8 pmol/L	T3 4.305 ± 1.01 pmol/L	0.0012
	T4 24.7 ± 1.9 pmol/L	T4 18.7 ± 2.4 pmol/L	0.0462

TSH—thyroid-stimulating hormone; T4—thyroxine; T3—3,5,3’-triiodothyronine.

**Table 2:** Association between genotypes and clinical outcome. *p*-values are estimated by exact test of Cochran—Armitage trend test across genotypes.

Gene	Reference SNP	SNP	Ancestral Allele	No. of Patients		*p*-Value	q-Value
				AIT2	Control		
NIS (SLC5A5)	rs7250346	C/G	C	16	17	0.5579	0.9679
		C/C		23	18		
		G/G		0	4		
TPO	rs732609	C/C	A	8	4	0.7414	0.9679
		A/C		14	24		
		A/A		17	11		
TSHR	rs1991517	C/C	C	25	29	0.4617 *	0.9066
		C/G		14	10		
GPX3	rs8177412	T/T	T	29	31	0.3808	0.9679
		C/C		1	3		
		C/T		9	5		
GPX4	rs713041	T/T	C	5	10	0.1892	0.7696
		C/C		12	11		
		C/T		22	18		
DUOX1-1 **	rs2458236	T/T	T	14	14	0.6101	0.9679
		C/C		6	10		
		C/T		9	15		
DUOX1-2 **	rs17595239	G/G	G	21	32	0.0063	0.0493
		G/T		17	7		
		T/T		1	0		
DUOX1-3 **	rs16939752	T/T	T	33	33	0.7449	0.9679
		C/T		6	6		

* *p*-values are estimated by chi-square with Yates’ correction. AIT2—type 2 amiodarone-induced thyrotoxicosis study group. **—Genes are labeled with additional number indexes for convenience; SNP—single nucleotide polymorphisms; AIT2—amiodarone-induced thyrotoxicosis 2 type; NIS (SLC5A5)—(Na)-iodide symporter (solute carrier family 5 member 5); TPO—thyroid peroxidase; TSHR—thyroid stimulating hormone receptor; GPX3—glutathione peroxidase 3.

**Table 3:** The regression analysis for the genotype distribution.

Gene	Genotype	*p*-Value	OR	CI (95%)
NIS	C/G	0.0983	1.386	0.795–2.411
	G/G	0.133	1.692	0.858–3.793
TPO	C/C	0.0724	1.127	0.737–2.009
	A/C	0.871	1.447	0.555–2.822
TSHR	C/G	0.4622	0.665	0.157–3.065
C/G	C/C	0.3313	0.590	0.218–2.430
	C/T	0.4351	1.397	0.823–4.255
GPX4	T/T	0.3482	0.728	0.211–1.095
	C/T	0.3382	0.771	0.105–3.202
DUOX1-1	C/C	0.4794	0.769	0.881–2.795
	C/T	0.5341	0.801	0.806–2.422
DUOX1-2	G/G G/T T/T	0.0131 0.0240 Not converged	0.2188 3.180	0.08863–0.4669 2.006–5.254
DUOX1-3	T/T	0.3332	0.695	0.1541–1.298
	C/T	0.4682	0.854	0.2035–3.172

*p*-values are estimated by binary logistic regression analysis. OR: Odds ratio, CI (95%): 95% Confidence interval. All SNPs are related to DUOX1 gene. Genes are labeled with additional number indexes for convenience. OR—odds ratio; CI—confidence interval; NIS—(Na)-iodide symporter; TPO—thyroid peroxidase; TSHR—thyroid stimulating hormone receptor; GPX3—glutathione peroxidase 3.

## Data Availability

Raw data supporting the conclusions of this publication will be made available by the authors without undue reservations.

## Supplementary Materials

The supporting information can be downloaded at: https://www.mdpi.com/article/10.3390/ijms24044016/s1.
