# Supplementary material for: DUOX1 Gene Missense Mutation Confers Susceptibility on Type 2 Amiodarone-Induced Thyrotoxicosis
Source: Int J Mol Sci. 2023 Feb 16;24(4):4016. doi: 10.3390/ijms24044016 (PMC9964217; doi:10.3390/ijms24044016)
Supplement: Supplementary file 1 [file ijms-24-04016-s001.zip › ijms-2107171-supplementary.pdf]

DUOX1 Gene Missense Mutation Confers Susceptibility on Type 2 Amiodarone-Induced Thyrotoxicosis

Olga Biakina [1,†], Yulia Mitina [2,3,†], Daria Gognieva [1,4], Marina Axenova [5], Alexandra Ermolaeva [6], Afina Bestavashvili [4,*], Valentin Fadeev [6], Abram Syrkin [1] and Philipp Kopylov [1,4]

Supplementary S1

List of primers and TaqMan probes using in DNA genotyping

rs7250346 NIS gene (replacement - C / G):

forward NIS primer: 5′-GCGTGCACAGAATTGCATAC-3 ′,

reverse NIS primer: 5'-TGTGAGTGGGGAAGAGAAGC-3 '.

NIS allele probe "G" 5'-R6G-CGCTCTGCCTCCAAAATTT-BHQ1-3 ',

NIS allele probe "C" 5'-FAM-CCCTCTGCCTCCAAAATTT-BHQ1-3 '.

Ta = 62 ° C

rs732609 TPO gene (replacement - G / T):

forward TPO primer: 5'-GGCAAATTCCCCGAAGACTT-3 ',

reverse TPO primer: 5'-GTGCTGTAACGTGGTGTGA-3 '.

TPO allele probe "T" 5'-R6G-TCACTGGCATGAACCTGG-BHQ1-3 ',

TPO allele probe "G" 5'-FAM-TCCCTGGCATGAACCTGG-BHQ1-3 '.

Ta = 60 ° C

rs1991517 TSHR gene (replacement - G / C):

forward TSHR primer: 5′-TCCTCCAAAGAACAGCACTGA-3 ′,

reverse TSHR primer: 5'-TTTGGCCTTGCTTCTTTGGG-3 '.

TSHR allele probe "G" 5'-R6G-GAGATGAGGCAGGGTCTC-BHQ1-3 ',

TSHR allele probe "C" 5'-FAM-GACATGAGGCAGGGTCTC-BHQ1-3 '.

Ta = 60 ° C

rs8177412 gene GPx3 (replacement - C / T):

forward GPx3 primer: 5'- CCTCGGGTGTGACCAATC -3 ',

reverse GPx3 primer: 5'- CCAGATTTGCTTATGTCACTGTC -3 '.

GPx3 allele probe "C" 5'-R6G- GGGACCCTTGCAGCCAA -BHQ1-3 ',

GPx3 allele probe "T" 5'-FAM- GAGACCCTTGCAGCCAA -BHQ1-3 '.

Ta = 62 ° C

rs713041 gene GPx4 (replacement - C / T):

forward GPx4 primer: 5′- CCCACTATTTCTAGCTCCACAAG -3 ′,

reverse GPx4 primer: 5'-GCTGGATTTTCGGGTCTG -3 '.

GPx4 allele probe "C" 5'-R6G- CTCGGAGCCTTCCACCG -BHQ1-3 ',

GPx4 allele probe "T" 5'-FAM- CTTGGAGCCTTCCACCG -BHQ1-3 '.

Ta = 64 ° C

rs2458236 gene Duox1-1 (replacement - C / T):

forward Duox1-1 primer: 5'- CAGAGAACCCAGAGCCTTGA -3 ',

reverse Duox1-1 primer: 5'-GGTGTCTCTTTGCTGTCCCT -3 '.

Duox1-1 allele probe "C" 5'-R6G- GAGCTCAGACCTGTGTGGA -BHQ1-3 ',

Duox1-1 allele probe "T" 5'-FAM-GAACTCAGACCTGTGTGGA -BHQ1-3 '.

Ta = 62 ° C

rs17595239 gene Duox1-2 (replacement - G / T):

straight Duox1-2 primer: 5′-AACAGTTCAGGCATATTTGCTCC-3 ′,

reverse Duox1-2 primer: 5'-CTGGGCCAAGGTCACAGTAGGT-3 '.

Duox1-2 allele probe "T" 5'-R6G-TTTTTCGTCACTTAATCTCCAGT-BHQ1-3 ',

Duox1-2 allele probe "G" 5'-FAM-TTGTTCGTCACTTAATCTCCAGT-BHQ1-3 '.

Ta = 60 ° C

rs16939752 gene Duox1-3 (replacement - C / T):

forward Duox1-3 primer: 5'- AGGCTAAGGCTTCCTGTCTC -3 ',

reverse Duox1-3 primer: 5'-GCCGGTAGTTCTCAATGAAG -3 '.

Duox1-3 allele probe "T" 5'-R6G- GCTGTCTCCACCAGACGGTG -BHQ1-3 ',

Duox1-3 allele probe "C" 5'-FAM- GCCGTCTCCACCAGACGGTG -BHQ1-3 '.

Ta = 62 ° C

Supplementary S2

List of primers

TPO

TPO-F: 5′- GGTTTTGGTGGGAGAACAGC -3′,

TPO-R408: 5′- GGCCTCCAGTTCCTTTCTCA -3′,

Ta=60C

NIS

NIS-F: 5′- GGTGAACAGGCAGAGAAGCA -3′,

NIS-R432: 5′- TCTCGACCACCATCATGTCC -3′,

Ta=60C

TSHR

TSHR-R: 5′- CTTCTGAGATTTGGCCTTGC -3′,

TSHR-F: 5′- CTCAGCAAGTTTGGCATCTG -3′,

Ta=60C

Duox1-1 (rs2458236)

Duox-1F: 5′- CTGAAGGGTCCCATCTGGAATT -3′,

Duox-1R: 5′- GAGCCTTGAACTCACAGTCAGGT -3′,

Ta=62C

Duox1-3 (rs16939752)

Duox-3F: 5′- CCACTGCCAGCTTGCTTATGC -3′,

Duox-3R: 5′- TAGGGACAGGGCTCGTCGTAAGTAA -3′

Ta=62C

PCR was performed with the following parameters: initial denaturation for 3 min at 94 ° C; then 33 cycles, including denaturation at 94 ° C for 10 s, annealing of primers at the indicated Ta for 10 s, synthesis of the chain at 72 ° C for 10 s, and final elongation at 72 ° C for 30 s.

PCR products were cut with appropriate restriction endonucleases (restriction enzymes), for which 25 µl of the amplificate was added to 2 µl of water, 3 µl of 10x appropriate restriction buffer, and the required amount of restriction enzyme, the mixture was incubated for 3 hours at the temperature optimal for each restriction enzyme. Separation of amplification and restriction products by size was carried out in a

horizontal 3% agarose gel. The genotype for each locus was determined based on the amplified DNA fragments and restriction products presented in Supplementary Table 1.

Table S1. Genotype determination for each locus based on amplified DNA fragments and restriction products.

Locus	PCR product length	Restrictase	Conditions	Length of the restriction product of the corresponding alleles
TPO, rs732609	408 bp	Bse1I 2U	65C, Buffer "Y"	A = 236+172 bp<br>C = 408 bp
NIS, rs7250346	432 bp	AccBSI 4U	37C, Buffer "Y"	C = 303+129 bp<br>G = 432 bp
TSHR rs1991517	191 bp	Fat I 5U	55C, Buffer "G"	C (727Asp) = 106+69+21 bp<br>G (727Glu) = 127+69 bp
Duox1-1 rs2458236	358 bp	AluI 1U	37C, Buffer "Y"	C = 44+32+184+98 bp<br>T = 44+32+282 bp
Duox1-3 rs16939752	413 bp	AluI 1U	37C, Buffer "Y"	C = 10 +403 bp<br>C = 10 +249+154 bp
